# *Leptospira* spp. infection in sheep herds in southeast Brazil

**DOI:** 10.1186/1678-9199-20-20

**Published:** 2014-05-06

**Authors:** Priscila Barbante, Fabio H Shimabukuro, Helio Langoni, Virgínia B Richini-Pereira, Simone B Lucheis

**Affiliations:** 1 Department of Veterinary Hygiene and Public Health, School of Veterinary Medicine and Animal Husbandry, São Paulo State University (UNESP – Univ Estadual Paulista), Botucatu, São Paulo State, Brazil; 2 Adolfo Lutz Institute, Sorocaba, São Paulo State, Brazil; 3 Adolfo Lutz Institute – CLR II, Bauru, São Paulo State, Brazil; 4 Paulista Agency of Agribusiness Technology (APTA/SAA), Av. Rodrigues Alves, 40-40, Bauru, SP, CEP 17030-000, Brazil

**Keywords:** Leptospirosis, Ovine, Serology, Culture, PCR

## Abstract

**Background:**

With the aim of studying *Leptospira* spp. infection in sheep herds, blood samples and respective kidney and liver fragments were collected from 100 animals from twenty different properties during slaughter at a meat company in the Sorocaba region, São Paulo state, southeast Brazil. The microscopic agglutination test (MAT) was performed with 29 strains of *Leptospira* spp. To identify the agent in the liver and kidney, 100 samples of each tissue were submitted to culture in Fletcher medium and analyzed by the polymerase chain reaction (PCR) for *Leptospira* spp.

**Results:**

MAT detected 23 samples serologically positive for one or more *Leptospira* spp. serovars and significantly more for Autumnalis. Eight (4%) samples were positive in culture (four kidneys and four livers), corresponding to five animals with positive serology (one animal simultaneously positive for both kidney and liver) and two negatives. PCR detected *Leptospira* spp. in 14 samples (seven kidneys and seven livers) corresponding to 12 positive animals (two animals simultaneously positive for kidney and liver), of which ten were serologically positive and two negative.

**Conclusions:**

PCR was faster, more practical and more sensitive than culture for detecting leptospires. The results reinforce the importance of sheep in the epidemiological context of leptospirosis.

## Background

One of the most representative zoonotic illnesses with a large economic impact on animal production is leptospirosis, an infectious disease that causes a fall in milk production, miscarriages, and low fertility. It is also a serious public health problem related to socioeconomic characteristics, floods, and occupational aspects in humans [1]. Azevedo *et al*. [2] were alerted to the transmission of the disease in slaughterhouse workers who had handled the organs and carcasses of infected animals.

*Leptospira* has been observed in the urine, semen and vaginal secretions of production animals, characterizing these species as susceptible to the disease from the reproductive sphere [3].

Ovine *Leptospira* spp. seems to be common in most countries, particularly in extensive flock management systems where sheep farming occurs together with cattle, allowing infection by direct contact with urine or by contaminated water in collective drinking supplies [4,5].

Infection of sheep was first detected in Brazil by Santa Rosa and Castro [6] in animals from São Paulo state, 34% of which were found to be reactive to various *Leptospira* spp. serovars.

Later serological studies performed by Viegas *et al*. [7], found 22.8% of sheep reactive, mainly to Autumnalis, Castellonis, Grippotyphosa, and Tarassovi serovars. In another work performed in Bahia, Caldas *et al*. [8] observed 34.7% of a sample of 800 examined sheep to be reactive, most frequently to the serovars Autumnalis, Castellonis and Butembo.

Langoni *et al*. [9] studied antileptospira agglutinins in 356 ovine serum samples from different regions of São Paulo state, finding the following prevalences: Icterohaemorrhagiae (51.25%); Castellonis (20.63%), Hardjo (19.36%); Bratislava (16.25%); Andamana and Wolffi (11.88%); Copenhageni (8.75%); Grippotyphosa (4.34%); Pomona (2.5%), and Tarassovi (0.63%).

A work performed by Martins and Lilenbaum [10] in Rio de Janeiro, Brazil, reported the highest prevalence of seroreactivity in sheep (47.4%), in relation to the other species of ruminants studied, including cows and goats.

In 11 municipalities in the Presidente Dutra microregion, Maranhão state, Brazil, Carvalho *et al*. [11] analyzed 379 sheep blood serum samples using a microscopic agglutination test (MAT); of the 37 herds studied, 30 (81%) had at least one seropositive animal while the individual seroprevalence was 32%.

A study performed in the state of Rio Grande do Sul verified by MAT that from 1360 tested serum samples, 466 (34.26%) animals were reactive with antileptospira agglutinin counts varying from 100 to 3200. The main serovars encountered were: Hardjo (28.4%), Sentot (16.8%); and Hardjoprajitno (14.5%), showing that *Leptospira* spp. is spread on most farms that raise sheep in the southeast and southwest mesoregions of Rio Grande do Sul [12].

A study by Escócio *et al*. [13] analyzed the sanitary profile of sheep flocks farmed exclusively or together with cattle in the Sorocaba region of São Paulo state. High levels of leptospirosis were found and all flocks were reactive to at least one serovar of *Leptospira* spp. Autumnalis was most prevalent serovar in four sheep flocks, followed by the Pyrogenes; in seven flocks where both cattle and sheep were in the same environment, the most prevalent were the serovars Icterohaemorrhagiae, Hardjo and Javanica.

Sheep flock infection by *Leptospira* leads to serious economic losses, represented by physiological problems and reproductive alterations. In this context, the aim was to verify the occurrence of antileptospira antibodies in sheep from different municipal areas in the Sorocaba region, São Paulo state, that were slaughtered in meat plants, to study the presence of serologically positive and negative sheep as renal *Leptospira* spp. carriers (chronic phase) and detect the agent in the liver (acute phase) by culturing in Fletcher medium using the Pasteur pipette technique and by PCR.

## Methods

### Animals and samples

We sampled 100 sheep from 20 properties from different municipal areas of Sorocaba region, São Paulo state, southeast Brazil. Samples were taken at moment of slaughter, selecting five animals from each property.

During the bleeding phase, approximately 10 mL of blood was collected from each animal in a 15 mL sterile glass tube. After coagulant removal, samples were centrifuged at 3000 rpm for 15 minutes and the serum obtained was treated in a 1.5 mL microtube and frozen at –20°C for later serological testing.

Fragments from the liver and the left or right kidney were aseptically collected during the disembowelment phase. These were placed individually in plastic bags, sealed and identified, refrigerated in an isothermal box, and transferred to the laboratory.

### Microscopic Agglutination Test (MAT)

MAT was performed as per Ministry of Health norms [14]. Each serum sample was initially diluted 1:100 in pH 7.2 phosphate buffered saline (PBS) as a positive cutoff point. Live cultures of 29 strains of *Leptospira* spp., used as antigens, were grown in liquid culture medium of Ellinghausen-McCullough-Johnson-Harris (EMJH), free of contamination or self-agglutination: *L. interrogans* serovars Australis, Autumnalis, Bataviae, Bratislava, Canicola, Copenhageni, Djasiman, Wolffi, Icterohaemorrhagiae, Pomona, Sentot, Hardjo, Hardjoprajitno, Hardjobovis, HardjoCTG and Hardjominiswajezak; *L. santarosai* serovar Shermani; *L. borgpetersenii* serovars Castellonis, Hebdomadis, Javanica, Pyrogenes, Tarassovi and Whitcombi; *L. kirschneri* serovars Butembo, Cynopteri and Grippotyphosa; *L. noguchi* serovar Panama and *L. biflexa* serovars Andamana and Patoc.

Considered positive were those serovars that presented 50% or more agglutination than the control. Samples found positive in the first titer were successively re-diluted at 1:2 and tested for the previously reacting serovars. The last titer was that which still presented 50% or more agglutination [15].

### *Leptospira* spp. isolation

The liver and kidney fragments used for culture were processed as cited by Passos *et al*. [16]. The obtained material was cultivated in three tubes with Fletcher culture medium, two with an additional 100 μg 5-fluorouracil/mL and 2.5 μg neomycin, and one without antibiotics, all incubated at 29°C for 16 weeks. Readings were made fortnightly after seeding; samples were considered positive when mobile spirochetes were seen under a dark field microscope at 400×.

### Polymerase Chain Reaction (PCR)

Fragments of liver and kidney weighing between 5 and 50 mg were pre-titrated with the aid of sterile pincers and bistoury and placed in sterile 1.5 mL DNAse and RNAse-free microtubes. After 1 mL of pH7.2 sterile PBS was added, the samples were centrifuged at 19,000 g for 15 minutes (kidney) and 19,000 g for 30 minutes (liver) at 4°C according to Heinemann *et al*. [17] with some modifications for tissue wash; supernatant was discarded and 50 μL of pH 7.2 sterile PBS was added to the cellular sediment, which was then macerated with a bio-vortexer (Biospec Inc., USA) and centrifuged at 2000 g for ten seconds.

The DNA extraction from liver and kidney was performed using an illustra Tissue & Cells Genomic Prep Mini Spin Kit (GE Healthcare, USA), as per the manufacturer’s recommendations and concentration was measured in a spectrophotometer (NanoVue, GE Healthcare, USA).

Molecular detection was carried out through PCR by using the primer pair: LEP1 (5′GGCGGCGCGTCTTAAACATG3′) and LEP2 (5′TTCCCCCCATTGAGCAAGATT3′), that amplified 331 bp for *Leptospira* spp. [18].

PCR reactions was performed in 0.2 mL microtubes with total volumes of 25 μL, containing PCR buffer solution (50 mM KCl, 10 mM of Tris-HCl pH 8.0), MgCl_2_ (1.5 mM), dNTP solution (0.2 mM), Taq Platinum DNA (1.0 U) (Invitrogen, Brazil), 10 ρM of each primer, ultra-pure water (Life Technologies, USA) and DNA (10 ng).

Amplification was performed in a Mastercycler® EP Gradient Thermal Cycler (Eppendorf, Germany). Thermal cycling conditions were 94°C for three minutes, 30 cycles of 94°C for one minute, annealing at 63°C for one minute, and extension at 72°C for two minutes, with an additional ten minutes at 72°C at the end to complete extension of the amplified segments. Visualization of amplified products was accomplished by electrophoresis. For this 1.5% agarose gel was prepared with 1 μL/mL SYBR® Safe DNA gel stain (Life Technologies, USA). PCR product (10 μL) and 4 μL of 100 bp molecular ladder (Life Technologies, USA) were used. To all samples, 2 μL of a BlueJuice™ Gel Loading Buffer (Life Technologies, USA) was added. The gel was submitted to the electrophoresis run in an HE99 horizontal cube (GE Healthcare, USA) containing 1X TBE (0.1 M Tris, 0.09 M boracic acid, and 0.001 M EDTA) at 100 V for approximately one hour using an electrophoresis power supply (EPS 301, GE Healthcare, USA). The gel was visualized in a UV transilluminator and the image captured by a GelDoc-It® TS Imaging System (UVP, USA) and documented using VisionWorks® LS Image Acquisition and Analysis Software (UVP, USA). Controls were used for extraction and PCR technique. Contaminated liver and kidney suspensions were prepared with *L. interrogans* serovar Pyrogenes at 2.0 × 10^4^ leptospires/mL as the positive control, and ultrapure water (Life Technologies, USA) as the negative control.

Analytical sensitivity was tested using different known negative tissue samples (liver and kidney) by contaminating the samples with *L. interrogans* serovar Pyrogenes at concentrations of approximately 2.0 × 10^0^; 2.0 × 10^1^; 2.0 × 10^2^; 2.0 × 10^3^, and 2.0 × 10^4^ microorganisms per milliliter of suspension from each organ sample [19].

## Results

From the 20 properties studied, 13 (65%) presented at least one seropositive animal. From the total 100 serum samples analyzed by MAT, 23 (23%) were reactive to one or more *Leptospira* spp. serovar.

Of the 29 *Leptospira* spp. serovars tested, only nine were reactive. Most animals were reactive to the Autumnalis serovar (n = 19) with titers varying from 100 to 1600. Other reactive serovars were Patoc (n = 3), Butembo (n = 2), Castellonis (n = 1), Djasiman (n = 1), Grippothyphosa (n = 1), Icterohaemorrhagiae (n = 1), Wolffi (n = 1) and Hardjo Prajitno (n = 1), whose titers varied from 100 to 200. The highest titer obtained was 1600 for Autumnalis serovar in a single animal (Table 1).

**Table 1:** **Distribution of *Leptospira *** **spp. serovars and antibody titers from sheep herds in Sorocaba region, São Paulo state, southeast Brazil**

**Property**	**Animals analyzed/positive**	**Serovars**											
		**Autumnalis**			**Butembo**	**Castellonis**	**Djasiman**	**Grippothyphosa**	**Icterohaemorrhagiae**	**Wollfi**	**Patoc**		**Hardjoprajitno**
		**100**	**200**	**1600**	**100**	**100**	**100**	**100**	**200**	**200**	**100**	**200**	**100**
**A**	5/1	1	–	–	–	–	–	–	–	–	–	–	–
**B**	5/0	–	–	–	–	–	–	–	–	–	–	–	–
**C**	5/1	1	–	–	–	–	–	–	–	–	–	–	–
**D**	5/2	1	1	–	–	–	–	–	–	–	–	–	–
**E**	5/2	1	1	–	–	–	–	–	–	–	–	–	–
**F**	5/1	1	–	–	–	–	–	–	–	–	–	–	–
**G**	5/2	1	1	–	–	–	–	–	–	–	–	–	–
**H**	5/2	1	1	–	–	–	–	–	–	–	–	–	–
**I**	5/0	–	–	–	–	–	–	–	–	–	–	–	–
**J**	5/1	–	1	–	–	–	–	–	–	–	–	–	–
**L**	5/3^a^	1	2	–	1	–	–	–	–	–	–	–	–
**M**	5/3^b^	1	1	–	–	–	–	–	–	1	–	1	–
**N**	5/0	–	–	–	–	–	–	–	–	–	–	–	–
**O**	5/0	–	–	–	–	–	–	–	–	–	–	–	–
**P**	5/0	–	–	–	–	–	–	–	–	–	–	–	–
**Q**	5/1	–	–	–	–	–	1	–	–	–	–	–	–
**R**	5/1	–	1	–	–	–	–	–	–	–	–	–	–
**S**	5/0	–	–	–	–	–	–	–	–	–	–	–	–
**T**	5/0	–	–	–	–	–	–	–	–	–	–	–	–
**U**	5/3^C^	1	–	1	1	1	–	1	1	–	1	–	1
**Total**	100/23	10	9	1	2	1	1	1	1	1	1	1	1

^a^One animal reactive to two serovars, Autumnalis (200) and Butembo (100).

^b^One animal reactive to two serovars, Autumnalis (200) and Patoc (200).

^c^One animal reactive to two serovars, Autumnalis (100) and Castellonis (100) and another to five serovars, Autumnalis (1600), Butembo (100), Grippothyphosa (100), Icterohaemorrhagiae (200), and Patoc (100).

(–) non-reactive animals.

In the culture results, from the 20 properties studied, we found five (25%) positive from at least one animal. Of the liver and kidney samples from 100 animals submitted to culture in Fletcher medium, four liver samples (three with and one without antibiotic) and four kidney samples (three with and one without antibiotic) were positive for *Leptospira* spp., corresponding to seven animals. Only one animal presented simultaneous positivity in both organs. Microorganisms were observed in the culture after one month of incubation at 29°C, confirmed by visualization of spirochetes in a dark field microscope. No opalescence ring formation was seen (Dinger zone).

PCR results from the 20 properties studied revealed that eight (40%) were positive for *Leptospira* spp. in at least one animal. In liver and kidney samples from 100 animals submitted to PCR, seven liver and seven kidney samples were positive for *Leptospira* spp., corresponding to 12 animals. Two sheep were positive for both kidney and liver samples. The analytical sensitivity was 2.0 × 10^2^ leptospires/mL.

Out of the 20 properties analyzed, 13 (65%) were reactive by at least one of the diagnostic tests used. Twenty-three animals were reactive by MAT, seven were positive in the Fletcher medium culture, and twelve were positive by PCR (Table 2).

**Table 2:** Results for microscopic agglutination test (MAT), culture and PCR of liver and kidney from sheep herds in Sorocaba region, São Paulo state, southeast Brazil

**Culture**		**MAT**	**PCR**		**Number of animals**
**Liver**	**Kidney**		**Liver**	**Kidney**	
–	–	–	–	–	75
–	–	+	–	–	13
–	–	+	–	+	3
–	+	+	–	+	1
–	+	–	–	+	1
–	+	+	+	+	1
+	+	+	+	+	1
+	–	–	+	–	1
–	–	+	+	–	2
+	–	+	+	–	2

+ positive; – negative.

## Discussion

Due to the paucity of data on the prevalence of leptospirosis in sheep flocks from different municipalities of the state of São Paulo, this study aimed to verify the occurrence of *Leptospira* spp. in different properties of the Sorocaba region, São Paulo state, using slaughterhouses as a strategic sample collection point. Thus, it was possible to establish the occurrence of leptospirosis in the flocks studied and highlight the most frequent serovars of *Leptospira* spp. from these regions.

According to serological results, 65% of the properties had positive animals, indicating that leptospirosis is present in the majority of sheep flocks in the Sorocaba region, thus demonstrating the importance of this disease in these animals.

The positive serology found in this study can be linked to previous contact with the etiological agent without disease development until the presence of ill carrier animals [20].

The most important probable infecting serovar was Autumnalis. This result corroborates the investigation by Viegas *et al*. [7] and Caldas *et al*. [8] in Bahia. This contrasts with Herrmann *et al*. [12], who reported Hardjo as the most prevalent serovar in sheep in Rio Grande do Sul state and Lilenbaum *et al*. [21], who observed that the most important serovars in Rio de Janeiro were Hardjo and Shermani.

Autumnalis is commonly isolated in wild animals, especially rodents, which could indicate them as possible leptospirosis transmitters in the sheep flocks studied [22]. This hypothesis is supported by the form of extensive farming observed in the present study.

Other probable infecting serovars were Djasiman, Hardjoprajitno, Wolffi, and Patoc, each reactive in only one animal. Wolffi and Hardjoprajitno are linked to the infection in bovines, Djasiman is found in wild animals, whereas Patoc is observed as a saprophytic serovar [23].

Some studies have demonstrated certain evidence that sheep are maintenance hosts for Hardjo, serving as a reservoir for bovines [24]. However, only one animal in this study demonstrated probable infection from this serovar.

Most reactive animals presented low antileptospirosis antibody titers, probably due to prior contact with the antigen. Only one animal presented a high titer of 1600 for Autumnalis with co-agglutination for other serovars, thus presenting serological reaction characteristic of acute infection [20].

Of the *Leptospira* spp. cultures from 100 renal and 100 hepatic samples cultured in Fletcher medium with and without antibiotic, eight were positive (four kidney and four liver) corresponding to five animals with positive serology (one was simultaneously positive in both kidney and liver) and two negatives. The isolation rate was 4% (8/200), better than values obtained by Azevedo *et al*. [2].

The low *Leptospira* spp. isolation rate could be dependent on various factors including type of medium used, serovar involved, sample processing time, type of material collected from the aseptic form, contamination, and selection of antibiotics used [25].

Failure to isolate the agent in the other 18 serologically positive animals could be explained by dealing with animals that had been in contact with a *Leptospira* without evolution of infection or disease or even by the factors cited by Thiermann *et al*. [25].

On the other hand, two serologically negative animals presented positive isolation, one for the kidney and the other for the liver. This exposes two probable scenarios: for the liver, the animal was in the initial infection phase without presenting MAT-detectable antibody titers; and in the kidney, the animal may have been infected by a different serovar than those tested in MAT without there being a cross-reaction.

Although this is a laborious, slow high-cost technique requiring months for results with little success in isolating the agent, identifying the serovar is highly important in epidemiological studies, as the isolated agent can be studied later to improve pathogen characterization and study its pathogenicity and other relevant analyses.

The presence of viable leptospires in the liver and kidneys of apparently healthy sheep at slaughter adds weight to the possibility of the disease being transmitted to the slaughterhouse and to meat packers who handle these materials. Furthermore, animal renal carriers could transmit the agent to rural workers from direct contact with urine or the contaminated environment.

The PCR of 100 renal and 100 kidney samples detected leptospires in 7% (14/200) samples (seven kidney and seven liver) corresponding to 12 positive animals (two animals were simultaneously positive for kidney and liver). Of the 23 serologically positive animals, leptospires were detected in ten while two did not present detectable antibodies by MAT.

Although PCR detected a higher positive sample rate than the culture technique, 13 serologically positive animals were negative. This can also be explained by what happened in culture, namely the absence of leptospire isolation in 18 seropositive sheep, probably because the animals had contact with the agent; but there was no progression of the disease.

The PCR technique was more practical and faster in detecting *Leptospira* spp. than culture in Fletcher medium and has the possibility of improving the sensitivity of diagnostic techniques in the early phase of infection and/or disease [19].

MAT is the worldwide reference test for leptospirosis diagnosis with high sensitivity and specificity. However, some difficulties with interpretation exist: there is a limitation in establishing whether serologically positive animals really are infected. Also, verification of a cross-reaction between different serovars can impede the establishment of the infecting serovar.

The presence of antileptospire antibodies often does not reflect the current situation of infection or the disease in animals, but rather only establishes host immunological response, which could be from prior contact without development of infection or the disease. This can be verified in the present study by the large number of serologically leptospire positive and negative animals from culture or PCR. This is reinforced by the weak concordance between serology and the agent’s presence in studied tissue.

In comparing the techniques and their concordance proportion, results from culture and PCR techniques in both tissues (liver and kidney) presented a good concordance (Kappa of 0.97). But when the results are analyzed only by looking at positive samples, of the seven positive liver and kidney samples by PCR, three were negative in both tissues in culture, corresponding to 42.8% negativity; in other words culture detected 42.8 times less than PCR. In addition, the sensitivity results from the two techniques for detecting the agent, taking serologically positive animals as the basis, showed PCR to be more sensitive than culture. This has also been found in other comparison studies [18].

The results from the three diagnostic techniques shows the probable stages of infection in the sheep, in agreement with Levett [20]. Seventy-five animals were negative in all tests performed, indicating absence of infection; thirteen animals were positive only in serology, probably indicating prior contact with the etiological agent without disease development, or an animal after the convalescence phase that is no longer a renal carrier, but still presents detectable antibodies (immunological memory). Four animals were serologically positive with kidney culture and/or PCR positive, which probably indicates chronic infection and renal colonization stage (renal carrier) with detectable antibodies. Four serologically positive animals that had liver culture and/or PCR positive were probably found in the acute infection phase, in leptospiremia and with detectable antibodies, but still without renal colonization. Two positive animals by both liver and kidney culture and/or PCR were possibly in the end acute stage, still with leptospiremia, detectable antibodies, and renal colonization. One animal was serologically negative with kidney culture and/or PCR positive, which suggests an infection by a serovar other than those used in the battery of antigens in MAT, it did not present cross-reaction to the tested serovars, and was found in the convalescence and renal carrier phase. Another serologically negative animal with positive liver culture and/or PCR probably had a recent infection, with leptospiremia, but still without detectable antibodies or renal colonization.

According to the probable infection stage found in animals with positive hepatic culture and/or PCR, seven sheep (30.4%) from the 23 serologically positive animals presented acute phase infection, which could indicate that infection is persistent on these properties.

## Conclusions

The occurrence of *Leptospira* spp. among sheep herds from Sorocaba region, state of São Paulo, Brazil, was 23% whereas the most important probable infecting serovar was Autumnalis, as determined by MAT. PCR was faster, more practical and sensitive than culture for detecting *Leptospira* spp. Our results reinforce the importance of sheep in the epidemiological context of leptospirosis in Brazil.

### Ethics committee approval

The present study was approved by the Ethics Committee on Animal Experimentation of the School of Veterinary Medicine and Animal Husbandry, São Paulo State University (UNESP), Botucatu, SP, Brazil, under protocol number 95/2008.

## Competing interests

The authors declare that there are no competing interests.

## Authors’ contributions

PB participated in the design of the study, carried out the blood collection, performed the diagnostic tests, the analysis of the results and took part in the article writing. FHS and HL participated in the analysis of the results. VBRP participated in the analysis of the results and article writing. SBL participated in the design of the study, the analysis of the results and article writing. All authors read and approved the final manuscript.

## References

[B1] Corrêa WM, Corrêa CNM Leptospiroses Enfermidades infecciosas dos mamíferos domésticos 1992 Rio de Janeiro: Medsi 219 231

[B2] Azevedo SS Alves CJ Andrade JSL Santos FA Freitas TD Batista CSA Isolation of *Leptospira* spp. from kidneys of sheep at slaughter Arq Inst Biol São Paulo 2004 71 3 383 385

[B3] Lilenbaum W Varges R Brandão FZ Cortez A de Souza SO Brandão PE Richtzenhain L Vasconcellos SA Detection of *Leptospira* spp. in semen and vaginal fluids of goats and sheep by polymerase chain reaction Theriogenology 2008 69 7 837 842 10.1016/j.theriogenology.2007.10.027 18291518

[B4] Ellis WA Bryson DG Neill SD McParland PJ Malone FE Possible involvement of leptospires in abortion, stillbirths and neonatal deaths in sheep Vet Rec 1983 112 13 291 293 10.1136/vr.112.13.291 6845608

[B5] Ellis GR Partington DL Hindmarsh M Barton MD Seroprevalence to *Leptospira interrogans* serovar *hardjo* in merino stud rams in South Australia Aust Vet J 1994 71 7 203 206 10.1111/j.1751-0813.1994.tb03402.x 7945098

[B6] Santa Rosa CA Castro AFP Presença de aglutininas anti-leptospira em soros de ovinos e caprinos no Estado de São Paulo Arq Inst Biol São Paulo 1963 30 93 98

[B7] Viegas EA Viegas SARA Caldas EM Aglutininas anti-leptospira em hemo-soro de caprinos e ovinos no Estado da Bahia Arq Esc Med Vet Univ Fed Bahia 1980 5 1 20 34

[B8] Caldas EM Sampaio MB Viegas EA Viegas SARA Dias EMM Aglutininas antileptospira em ovinos e caprinos na região Nordeste do Estado da Bahia Arq Esc Med Vet Univ Fed Bahia 1983 8 1 88 98

[B9] Langoni H Marinho M Baldini S Silva AV Cabral KG Silva ED Pesquisa de aglutininas anti-leptospiras em soros ovinos do Estado de São Paulo, Brasil, utilizando provas de macroaglutinação em placa e soroaglutinação microscópica Rev Bras Med Vet 1995 17 6 264 268

[B10] Martins G Lilenbaum W The panorama of animal leptospirosis in Rio de Janeiro, Brazil, regarding the seroepidemiology of the infection in tropical regions BMC Vet Res 2013 9 237 doi:10.1186/1746-6148-9-237. [http://www.biomedcentral.com/1746-6148/9/237] 10.1186/1746-6148-9-237 24289165 PMC4220826

[B11] Carvalho SM Mineiro AL Castro V Genovez ME Azevedo SS Costa FA Leptospirosis seroprevalence and risk factors for sheep in Maranhão state, Brazil Trop Anim Health Prod 2014 46 2 491 494 10.1007/s11250-013-0505-1 24326771

[B12] Herrmann GP Lage AP Moreira EC Haddad JPA Resende JR Rodrigues RO Leite RL Soroprevalência de aglutininas anti-*Leptospira* spp. em ovinos nas Mesorregiões Sudeste e Sudoeste do Estado do Rio Grande do Sul, Brasil Ciênc Rural 2004 34 2 443 448

[B13] Escócio CF Genovez ME Castro V Paulin LMS Piatti RM Okuda LH Gabriel FHL Chiebao DP Felicio PS Almeida MCS Perfil sanitário de rebanhos ovinos criados exclusivamente ou consorciados com bovinos na região de Sorocaba - São Paulo 35° Congresso Brasileiro De Medicina Veterinária: 19-22 Out 2008, Gramado, RS 2008 Gramado: Conbravet [http://www.sovergs.com.br/conbravet2008/anais/cd/resumos/R0693-2.pdf]

[B14] Brasil. Ministério da Saúde. Secretaria de Vigilância em Saúde Guia Leptospirose: Diagnóstico e Manejo Clínico. Ministério da Saúde, Secretaria de Vigilância em Saúde, 2009. Em ftp://ftp.cve.saude.sp.gov.br/doc_tec/ZOO/LEPTO09_GUIA_MANEJO.pdf

[B15] World Health Organization Guidelines for the control of leptospires 1982 Geneva: WHO Offset Publication

[B16] Passos EC Vasconcellos SA Ito FH Yasuda PH Nürmberger Junior R Isolamento de leptospiras a partir do tecido renal de hamsters experimentalmente infectados com *Leptospira interrogans* sorotipo *pomona*. Emprego das técnicas da pipeta Pasteur e a das diluições seriadas em meios de cultura de Fletcher tratado com 5-fluor-uracil ou o sulfato de neomicina Rev Fac Med Vet Zootec Univ São Paulo 1988 25 2 221 235 10.11606/issn.2318-3659.v25i2p221-235

[B17] Heinemann MB Garcia JF Nunes CM Gregori F Higa ZM Vasconcellos SA Richtzenhain LJ Detection and differentiation of *Leptospira* spp. serovars in bovine semen by polymerase chain reation and restriction fragment length polymorphism Vet Microbiol 2000 73 4 261 267 10.1016/S0378-1135(00)00150-4 10781725

[B18] Mérien F Amouriaux P Perolat P Baranton G Saint-Girons I Polymerase chain reaction for detection of *Leptospira* spp. in clinical samples J Clin Microbiol 1992 30 9 2219 2224 1400983 10.1128/jcm.30.9.2219-2224.1992 PMC265482

[B19] Shimabukuro FH Domingues PF Langoni H Silva AV Pinheiro JP Padovani CR Pesquisa de suínos portadores renais de leptospiras pelo isolamento microbiano e reação em cadeia pela polimerase em amostras de rins de animais sorologicamente positivos e negativos para leptospirose Braz J Vet Res Anim Sci 2003 40 4 243 253

[B20] Levett PN Leptospirosis Clin Microbiol Rev 2001 14 2 296 326 10.1128/CMR.14.2.296-326.2001 11292640 PMC88975

[B21] Lilenbaum W Varges R Ristow P Cortez A Souza SO Richtzenhain LJ Vasconcellos SA Identification of *Leptospira* spp. carriers among seroreactive goats and sheep by polymerase chain reaction Res Vet Sci 2009 87 1 16 19 10.1016/j.rvsc.2008.12.014 19232418

[B22] da Silva RC Zetun CB Gimenes Bosco SM Bagagli E Rosa PS Langoni H Toxoplasma gondii and *Leptospira* spp. infection in free-ranging armadillos Vet Parasitol 2008 157 3–4 291 293 18809258 10.1016/j.vetpar.2008.08.004

[B23] Salaberry SRS Epidemiologia das principais doenças infecciosas de ovinos do município de Uberlândia, MG Thesis 2010 Faculdade de Medicina Veterinária, Universidade Federal de Uberlândia

[B24] Gerritsen MJ Koopmans MJ Peterse D Olyhoek T Sheep as maintenance host for *Leptospira interrogans* serovar *hardjo* subtype *hardjobovis* Am J Vet Res 1994 55 9 1232 1237 7802389

[B25] Thiermann AB Leptospirosis: current developments and trends J Am Vet Med Assoc 1984 184 6 722 725 6725107

